# The Influence of Sport Expertise on Response and Cognitive Inhibition

**DOI:** 10.1177/00315125261418880

**Published:** 2026-01-19

**Authors:** Jack Brimmell, Naomi Lee, Matt Spokes

**Affiliations:** 1 Department of Psychology and Mental Health, School of Health and Wellbeing, 8709 University of Worcester, Worcester, UK

**Keywords:** athletic expertise, cognitive inhibition, expertise-induced-amnesia, perceptual-cognition, response inhibition

## Abstract

Research suggests that sporting experts show better response inhibition. Less is known about how expert athletes use cognitive inhibition. Experts may cognitively inhibit, or ‘forget’, previous errors via an expertise-induced-amnesia which suggests that experts have impoverished episodic memory due to the reduced task attention when performing well-rehearsed actions. This amnesia may be relevant at low-pressure, but most interestingly may be more of a factor at high-pressure. The aim of the present study was to examine whether sporting expertise predicted response inhibition (effectiveness and efficiency) and cognitive inhibition (error awareness) at low-pressure and high-pressure, respectively. Forty-five participants from various sports (static, interceptive or strategic) completed a measure of sporting expertise and a modified Stop Signal Task under two pressure conditions (manipulated via divergent task instruction). Regression results suggested that expertise only significantly predicted response inhibition effectiveness and efficiency at high-pressure. Interestingly, error awareness at high- and low-pressure were independent of sporting expertise. Finally, change scores across pressure conditions were small and near zero for all participants, not just experts, suggesting that all individuals performed similarly across low- and high-pressure conditions. Sporting expertise appears to facilitate response inhibition at high-pressure but the exact methods in which experts ‘forget’ errors and maintain performance remains unknown.

## Introduction

Perceptual-cognitive processes are important for expert sport performance (Mann et al., 2007). The ability to successfully locate, interpret, and utilise sensory information from the current environment are perceptual skills that often allow experts to make better decisions compared to their novice counterparts (Williams & Krane, 1998). Consider a soccer player who must locate and track the location of numerous external artefacts (i.e., teammates, opponents, the ball) alongside having to coordinate their own body and motor actions to best support the team (Swann et al., 2015). In such a situation, the individual with greater expertise will often be equipped to perform better (i.e., experts outperform novices on cognitively demanding tasks; Klostermann & Moeinirad, 2020; Lebeau et al., 2016). Perceptual-cognition is a broad construct that encompasses a variety of processes including visual attention, anticipation, motor-planning, and executive function (EF). The area of EF has become popular over recent years with research examining the impact of inhibition (e.g., Hagyard et al., 2021), shifting (e.g., Vestberg et al., 2012), updating (e.g., Wood et al., 2016), and in more select cases, holistic EF models (i.e., inhibition, shifting, and updating together; Brimmell et al., 2021; Scharfen & Memmert, 2021), upon sport performance and sporting expertise.

Executive functions are believed to be a family of related, yet distinct, attentional processes that help regulate and direct behaviour (Zelazo & Carlson, 2012). Inhibition is perhaps the most researched EF in sport (e.g., Brevers et al., 2018; Brimmell et al., 2021; Ducrocq et al., 2016; Hagyard et al., 2021; Klostermann, 2020) and refers to the ability to resist internal or external distraction that is no longer task appropriate (Diamond, 2013). Theoretically, it has been proposed that when trying to measure EFs like inhibition both EF effectiveness and efficiency should be considered (Eysenck et al., 2007). That is, an individual who can successfully inhibit when required could be considered effective. But people vary in the number of resources (e.g., effort or time) required to be effective and those who can inhibit using fewer resources could be called efficient (Eysenck et al., 2007).

The relevance of inhibition for sport performers is still a contested subject with some research stating the evidence for a relationship is weak. For example, Beavan et al. (2020) concluded there was no relevance of inhibition in sport when looking for far-transfer effects from lab-based tasks to real-world sport. However, the notion that there is no importance of inhibition is likely inaccurate and an oversimplification with a wealth of evidence to suggest the contrary. For example, Fleddermann et al. (2023) found, in a sample of elite strategic-sport athletes, that those with greater expertise showed greater motor inhibition (stopping of hand movements) compared to those with lower expertise. To compound this, a recent scoping review from Simonet et al. (2023) provides the overall message that athletes outperform non-athletes on measures of inhibition. Research also attests to the positive effect of inhibition training upon gaze behaviour and tennis performance (Ducrocq et al., 2016) and the interaction between inhibition and visual attention in successful sport performance (Brimmell et al., 2021; Klostermann, 2020).

Although we have evidence that experts have greater inhibition (Hagyard et al., 2021) and that inhibition may, in part, aid sport performance (Brimmell et al., 2021), the underlying effect of improved inhibition remains unclear. Specifically, the exact functional purpose of inhibition in expert performers is less known. The underlying role of inhibition is unlikely to be isolated to supporting any specific single function or action and it is more likely that inhibition contributes across numerous areas of sport performance. This notion is consolidated by Diamond’s (2013) model that parses inhibition into a multi-component construct comprising related, but divergent, forms of inhibition. Specifically, and often neglected in sport research, inhibition is proposed in Diamond’s (2013) model to comprise cognitive inhibition (i.e., supressing thoughts or memories), response inhibition (i.e., withholding behavioural responses), and selective attention (i.e., orienting attention to a certain location at the expense of others).

Given that each inhibition component is somewhat unique, it is reasonable to suggest that each component could vary in its influence upon expert sport performance. Response inhibition is the most studied component of inhibition in sport and refers to an ability to stop planned or ongoing action when said action interferes with the current goal and is particularly important in volatile environments like sport (Brevers et al., 2018; Brimmell et al., 2021). Tasks used to assess response inhibition are usually domain-general and are used to give an indication about how well someone can stop a preplanned motor response (e.g., Stop Signal Task; Verbruggen et al., 2019). This may be particularly relevant in complex sports where the environment is constantly changing and certain motor actions can quickly become incorrect (e.g., soccer). Selective attention may be more consistently examined through eye-trackers in sport given that selective attention often requires the individual to choose between ignoring irrelevant stimuli or not (Diamond, 2013). Cognitive inhibition, referred to as inhibition in the service of safeguarding information within working memory (e.g., keeping irrelevant information out; Diamond, 2013), is currently untested, but it might aid sports performers through an enhanced ability to supress unwanted thoughts and memories (Diamond, 2013), potentially via an interference control mechanism that supports intentional forgetting (Anderson & Levy, 2009).

The idea that sporting experts can be “oblivious” or forget certain actions is not new. For example, expertise-induced-amnesia has been proposed as a method by which experts tend to avoid over-attending to well-rehearsed actions and instead perform them more automatically (Beilock & Carr, 2001). The consequence of this is that experts have been found to give impoverished episodic recollections of action despite greater procedural knowledge (Beilock & Carr, 2001). Though originally describing an ability to episodically forget motor control, recent work from Harris et al. (2019) suggested that this ability may extend to forgetting one’s own mistakes. Harris et al. (2019) outlined that forgetting a mistake may alleviate subsequent anxiety associated with making that mistake, especially during high-pressure. However, this idea remains untested experimentally and the mechanisms that allow one to “forget” their mistake is unknown. It may be that greater inhibition allows individuals to suppress or withhold thoughts surrounding their past errors, thus “forgetting” them and instead focusing on current performance. As a result, subsequent performance likely benefits and this effect may be greater at high-pressure (Harris et al., 2019).

The issue with not forgetting, or moving beyond prior errors, is that it can negatively influence subsequent performance by creating feelings of anxiety around the upcoming performance (Harris et al., 2019). This feeling of anxiety can be enhanced when pressure is high due to an increased sense of importance within the situation (Baumeister, 1984). Specifically, committing an error can pull attentional resources away from the current task and instead direct attentional focus toward the past mistake creating feelings of anxiety and increasing the perception that future errors will occur (Harris et al., 2019). At a higher level of pressure, experts may be better equipped, via an enhanced inhibition ability, to focus attention on the present task (goal-directed attention; Corbetta & Shulman, 2002) and avoid feelings of anxiety concerning past mistakes. There are numerous examples of sporting experts performing equivocally or better under pressure (e.g., Otten, 2009) and in heightened states of anxiety (e.g., Nibbeling et al., 2012) but again, no work has investigated the potential role of inhibition in helping experts move beyond errors. Understanding this has important implications in sports performance. For example, whilst experts forgetting errors due to greater inhibition may be beneficial in the moment by reducing anxiety, it may also hinder longer-term improvements through reduced accurate reflection. Therefore, training and coaching practices to correct errors may require an altered approach.

In sum, recent research is mixed with some doubting the importance of response inhibition for sport performers (e.g., Beavan et al., 2020) while others suggest response inhibition is key (e.g., Hagyard et al., 2021). In addition, it may be that experts are more able to move past or forget previous errors due to an enhanced cognitive inhibition ability (i.e., the ability to suppress thoughts and memories; Diamond, 2013). If this is accurate, and akin to how expertise-induced-amnesia models show experts have poorer episodic recollection of motor actions, it may be that those with greater expertise have poorer immediate error awareness. As these ideas remain unclear or untested, the aim of the present work was to assess whether sporting expertise predicted response inhibition (effectiveness and efficiency) and cognitive inhibition (error awareness) at low- and high-pressure, respectively. Finally, if experts are indeed able to maintain or improve performance under pressure (Harris et al., 2019; Otten, 2009), then their performance should be equivocal, or better, in high-pressure conditions compared to low-pressure conditions. The hypotheses were as follows:

H1 Higher sporting expertise is expected to lead to enhanced response inhibition effectiveness, response inhibition efficiency, and cognitive inhibition in the low-pressure condition.

H2 Higher sporting expertise is anticipated to lead to greater response inhibition effectiveness, response inhibition efficiency, and cognitive inhibition in the high-pressure condition.

H3 Higher sporting expertise should allow individuals to either perform comparably across the low- and high-pressure conditions or exhibit improved performance when transitioning from the low-pressure to the high-pressure condition. Therefore, higher sporting expertise should predict greater positive change scores.

## Method

### Participants

*A priori* power analysis in G*Power software (version 3.10) outlined that 42 participants would be needed. This number was based on previously reported medium effect sizes (e.g., *R*^*2*^ = .15; Hagyard et al., 2021) and detecting a medium effect size (f^2^ = .20), with power = .80, at an alpha value of .05, with one tested predictor (i.e., expertise). In total, 45 participants took part in the present study (M_age_ = 27.98 years ± 10.29years; Male = 23, Female = 21, Prefer not to say = 1). All participants were currently residing in the United Kingdom, though some (*n* = 6) provided sporting expertise information from a time playing elsewhere (i.e., Canada = 1, India = 2, Iran = 1, Syria = 1, and Ukraine = 1). Based on Krenn et al.’s (2018) classification, the type of sport participated in was considered either static (e.g., climbing, gym, running, swimming, and yoga; *n* = 19), interceptive (e.g., archery, badminton, cycling, dance, equestrian, tennis, trampolining, and mixed-martial arts; *n* = 18), or strategic (e.g., basketball, cricket, and football; *n* = 8). Informed consent was acquired from all participants before the experiment following procedures approved by a local ethics committee.

### Measures

#### Expertise

Expertise was calculated based on the framework proposed by Swann et al. (2015). An individual expertise score was based on participants responses to (A) highest performance level, (B) success at their highest level, (C) experience (in years) at their highest level, (D) competitiveness of individuals main sport in country of play, and (E) global competitiveness of the individual sport. Components A-E were given an individual score between 0–4 before being entered into the following equation – [(A + B + C/2)/3] × [(D + E)/2] – which provided an individual expertise score where higher scores represent higher expertise. The mean expertise score was 3.72 (±2.14) and scores ranged from .83 to 11.33. Following the classification recommendations in Swann et al. (2015) the present participants were classed as semi-elite (*n* = 30), competitive elite (*n* = 13), and successful elite (*n* = 2). This procedure has been successfully applied in previous research (Brimmell et al., 2021; Hagyard et al., 2021).

#### Inhibition

A modified version of the Stop Signal Task (SST; Verbruggen et al., 2019) was used to assess both response and cognitive inhibition (original script available on https://millisecond.com and the authors’ modified script is available at: https://osf.io/xg3vd/?view_only=81b1f402b28a42ca978b08ee0b97e164). The SST was made up of 75% “go” trials and 25% “stop-signal” trials. “Go” trials involved a centrally located arrow stimulus facing either left (requiring a “D” key response made with the index finger of the left hand) or right (requiring a “K” key response made with the index finger of the right hand). The “stop-signal” trials were the same as “go” trials but included a stop signal (i.e., an auditory beep). On these trials, participants aimed to provide no response (i.e., inhibit responses). To ensure all participants committed errors an adaptive procedure was used for the stop signal delay (as outlined in Verbruggen et al., 2019). After each successful inhibition (i.e., no response on a “stop-signal” trial) the delay increased by 50 ms and after each unsuccessful inhibition (i.e., a response on a “stop-signal” trial) the delay decreased by 50 ms with a minimum delay of 50 ms and maximum delay of 1150 ms (Verbruggen et al., 2019).

The outcome variables for response inhibition included performance effectiveness (i.e., number of correct responses on “stop-signal” trials [no response] minus number of incorrect responses on “stop-signal” trials [responding when not appropriate]) and efficiency (i.e., stop signal reaction time [SSRT]^1^; Verbruggen et al., 2019). High effectiveness scores reflect greater effectiveness while low efficiency scores outline greater efficiency. Therefore, it is predicted that greater sporting expertise will link to high effectiveness scores and low efficiency scores. For cognitive inhibition, the outcome variable was error awareness which was calculated as the percentage of errors on “stop-signal” trials recalled from total errors (i.e., [errors recalled/total errors made] * 100). To capture this, participants were informed at the beginning of the task to give double key presses on trials that they believed followed a ‘stop-signal’ trial error. That is, if the target arrow was facing left following a ‘stop-signal’ trial error they must press the “D” key twice to show awareness of this error. Following the expertise-induced-amnesia model low error awareness values are likely linked to higher sporting expertise. The task comprised two test blocks of 100 trials (one block of low-pressure trials and one block of high-pressure trials) as well as 10 practice trials.

#### Situational Stress

To assess whether divergent pressure conditions (i.e., high- and low-pressure) were successfully created (see ‘Pressure Manipulation’ below) situational stress was measured via the Stress Rating Questionnaire (SRQ; Edwards et al., 2015). The SRQ assesses five bipolar dimensions (e.g., calm to nervous) with each item answered on a 7-point Likert scale. The SRQ was administered before any task instructions were given (i.e., baseline-SRQ), and then again following each pressure-instruction set (i.e., low-pressure-SRQ and high-pressure-SRQ). Larger changes from baseline to post instruction indicate greater situational stress. The SRQ correlates with alternate established anxiety measures (e.g., the State-Trait Anxiety Inventory; Edwards et al., 2015), has been previously used to assess pressure instruction efficacy (Brimmell et al., 2021), and has satisfactory internal consistency (α = 87-.89; Brugnera et al., 2017).

### Pressure Manipulation

The present study was interested in inhibition performance at varying levels of pressure given the capacity for sporting pressure to fluctuate (Harris et al., 2019). In the low-pressure condition the task instructions focused on basic information sufficient to complete the task with the general notion that such trials could be considered ‘familiarisation’. This included information on which keys to hit to record a response and that the goal was to provide no response after the stop signal. The high-pressure condition instructions were designed to increase the perceived importance of such trials and imply that individuals would be evaluated based on their performance. These instructions were based off previous work eliciting a similar response in soccer penalties (Brimmell et al., 2021) and based off literary recommendations (Gropel & Mesagno, 2019). Specifically, participants were informed that this block of trials was the most important, responses must be as fast and accurate as possible, only their best efforts were acceptable, and that performance on these trials were to be evaluated and analysed by the researchers. The instruction to use double key presses to indicate error awareness was given in both low- and high-conditions.

### Procedure

Ethical approval was provided by the lead authors institution. For a visual overview of the procedure, see Figure 1. Initially, participants were welcomed to the private single-seater laboratory on university campus before reading an information sheet and providing written informed consent. Next, the self-report measures of age, gender, sporting expertise, and baseline-SRQ were completed. Participants then succesfully completed a single block of 10 practice trials before being informed about the first test block of the SST which was always the low-pressure condition. This choice was made given the emphasis on ‘practice’ in this block and to avoid any issues arising from placing a ‘practice’ block after a ‘serious’ test block. After receiving the low-pressure instructions, participants completed the low-pressure-SRQ and the first SST test block. A similar protocol was used for the second SST test block. That is, they received instructions, this time the high-pressure instructions, before completing the high-pressure-SRQ and the second SST test block. In total, participants completed two experimental blocks (one low-pressure, one high-pressure) each with 100 trials (comprised of 75 go trials and 25 “stop-signal” trials). All participants completed the SST on a Dell laptop with a 14-inch screen running Windows 11. Finally, participants were debriefed, thanked for their time (the entire testing procedure lasted around 40-min), and provided with the contact information for any relevant support networks that may be needed due to the elevated psychological pressure (e.g., Mind Charity).

**Figure 1.**
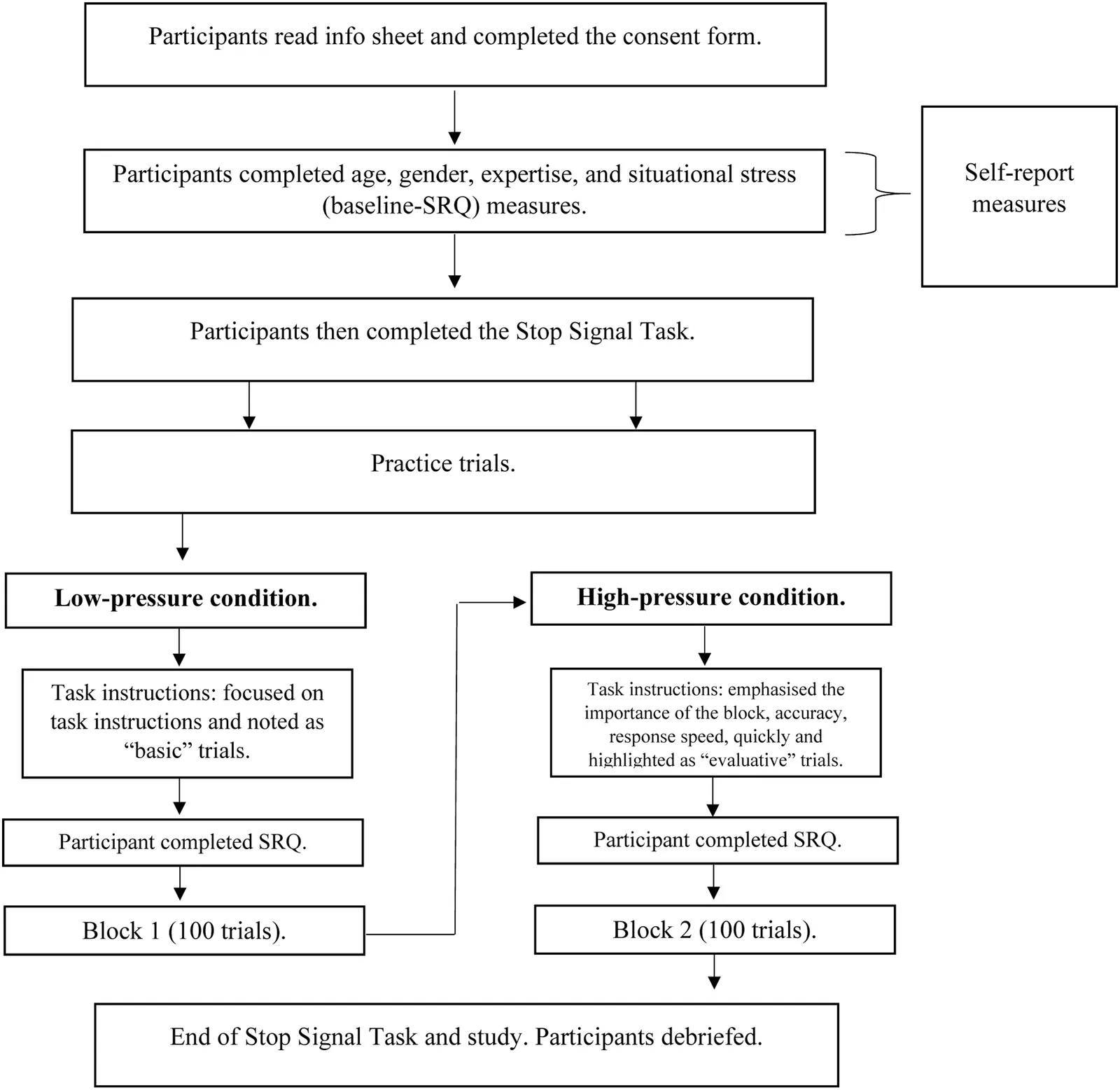
Method schematic diagram

### Design, Data Processing, and Data Analysis

The present study used an experimental cross-sectional design. Data pre-processing and analyses were performed in RStudio (version 4.0.2; R Core Team, 2023). All associated materials are available via the Open Science Framework: https://osf.io/xg3vd/?view_only=81b1f402b28a42ca978b08ee0b97e164. First, raw data pertaining to age, gender, sporting expertise, SRQ responses, and SST performance were converted to Excel files from Qualtrics (https://qualtrics.com) and Millisecond. Next, raw files were pre-processed in RStudio to create files for data analysis. The efficacy of the pressure manipulation instructions was assessed using within-subject *t*-tests. Specifically, to understand the impact of the low-pressure instructions we examined mean SRQ changes from baseline to post-low-pressure instruction. Similarly, to assess the effectiveness of the high-pressure instructions we examined mean SRQ changes from post-low-pressure instructions to post-high-pressure instructions. To assess H1, regression analyses were used where expertise was entered as a continuous independent variable and response (effectiveness and efficiency) and cognitive (error awareness) inhibition at low-pressure were the continuous dependent variables, respectively. For H2, a similar process was used but response (effectiveness and efficiency) and cognitive (error awareness) inhibition performance at high-pressure were the respective dependent variables.

To assess H3, change scores were calculated for each dependent variable. To create change scores, effectiveness, efficiency, and error awareness scores at low-pressure were subtracted from their corresponding score at high-pressure. These change scores were then entered as the dependent variable in regression models where sporting expertise was the predictor. To refer back to H3, change scores at or near zero suggest equivocal performance across low- and high-pressure conditions. Directional interpretation of the change scores was conditional to the variable. That is, positive change scores for effectiveness suggested improved performance in high-pressure vs low-pressure, negative efficiency change scores suggested improvements in the high-pressure condition compared to the low-pressure condition, and negative change scores for error awareness were indicative of a reduced percentage of errors recalled in the high-pressure condition compared to the low-pressure condition).

## Results

### Pressure Manipulation Check

Descriptive statistics for inhibition effectiveness, inhibition efficiency, and cognitive inhibition at both low- and high-pressure conditions are shown in Table 1. The SRQ scores could range from a minimum of five to a maximum of 35 (Edwards et al., 2015). The mean SRQ response at baseline was 11.38 (*SD* = 5.49), the mean SRQ response was 11.51 (*SD* = 5.43) after the low-pressure instructions, and the mean SRQ response was 13.09 (*SD* = 6.08) after the high-pressure instructions. The results of the *t*-test comparing baseline and low-pressure SRQ responses suggested a non-significant increase in SRQ responses (*t* (44) = .28, *p* = .785, Cohen’s *d* = .02). However, the results of the *t*-test comparing SRQ responses at post-low-pressure and post-high-pressure instructions outlined a significant difference (*t* (44) = 2.79, *p* = .008, Cohen’s *d* = .27). The results suggested that participants experienced a significant increase in self-reported stress and that the pressure manipulation was successful. This is corroborated by the non-significant increase from baseline to post-low-pressure instruction. See Figure 2 for an overview of SRQ responses at baseline, low-pressure, and high-pressure conditions.

**Table 1.** Shows descriptive statistics (i.e., mean and standard deviaition) for inhibition effectiveness, inhibition efficiency, and cognitive inhibition in both low- and high-pressure conditions.

	Low pressure	High pressure
Effectiveness, M (S.D)	−4.60 (12.30)	−6.02 (10.95)
Efficiency, M (_S.D_)	313.69 (194.54)	319.36 (201.00)
Error awareness, M (_S.D_)	61.65 (40.95)	67.60 (39.77)

**Figure 2.**
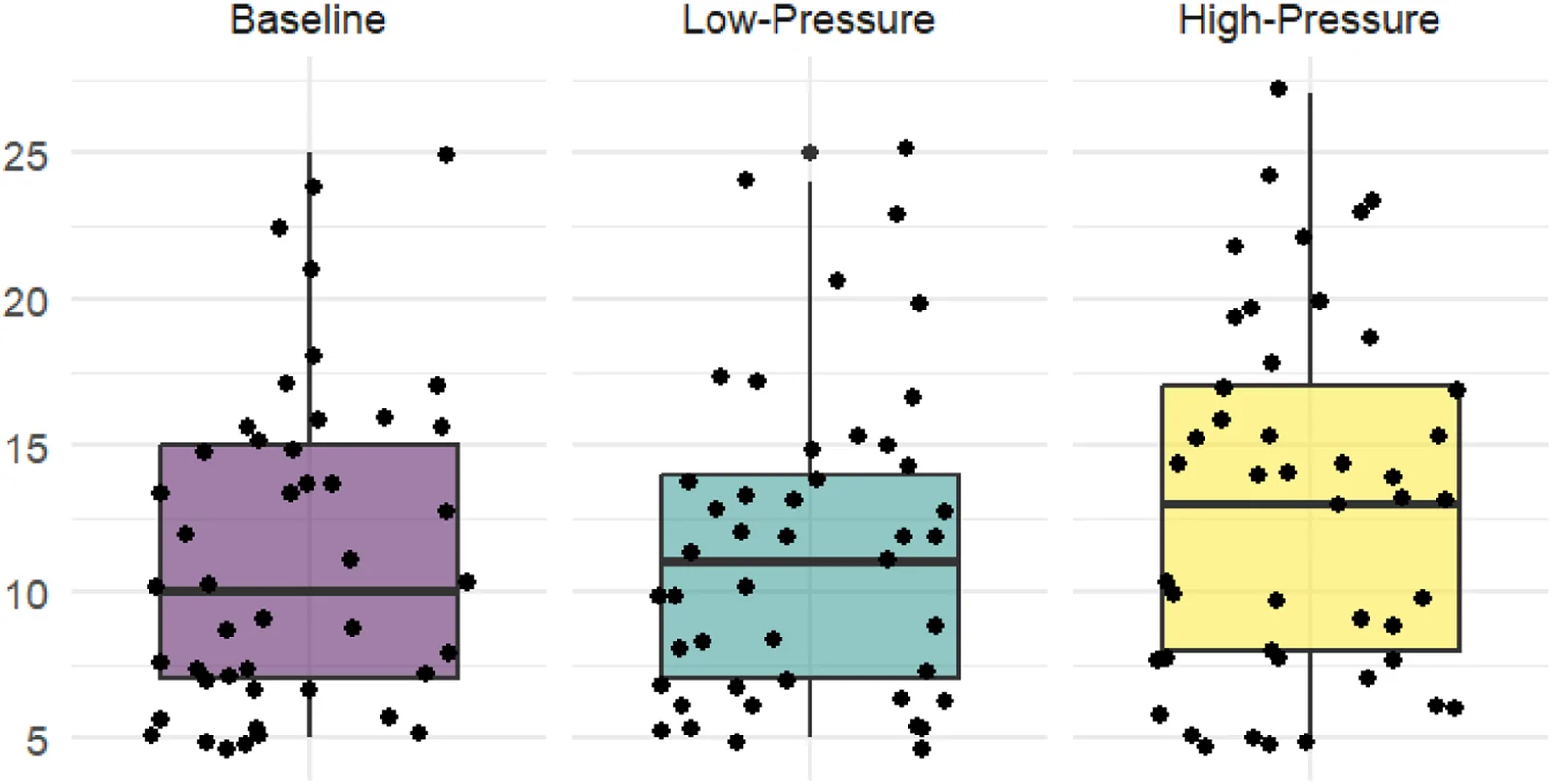
Individual stress rating questionnaire responses at baseline (left), post low-pressure instruction (middle), and post high-pressure instruction (right)

### Does Expertise Predict Greater Response Inhibition Effectiveness, Response Inhibition Efficiency, and Cognitive Inhibition (i.e., Error Awareness) at Low-Pressure (H1)

Regression results for the low-pressure condition suggested that expertise was not a significant predictor of response inhibition effectiveness (*F* (1, 43) = 2.61, *p* = .114, β = .24, 95% CI = [−.06, .54]; see Figure 3(a)) nor efficiency (*F* (1, 43) = 3.55, *p* = .066, β = −.28, 95% CI = [−.57, .02]; see Figure 3(b)), and the *R*^*2*^ values suggested that the models accounted for 5.7% (*R*^*2*^ = .057) and 7.6% (*R*^*2*^ = .076) of the variance, respectively. Expertise did not significantly predict cognitive inhibition (i.e., error awareness) in the low-pressure condition (*F* (1, 43) = .75, *p* = .391, β = .13, 95% CI = [−.17, .44]; see Figure 3(c)). The *R*^*2*^ values suggested that expertise accounted for 1.7% (*R*^*2*^ = .017) of the variance in cognitive inhibition within the low-pressure condition. These results are oppose H1.

**Figure 3.**
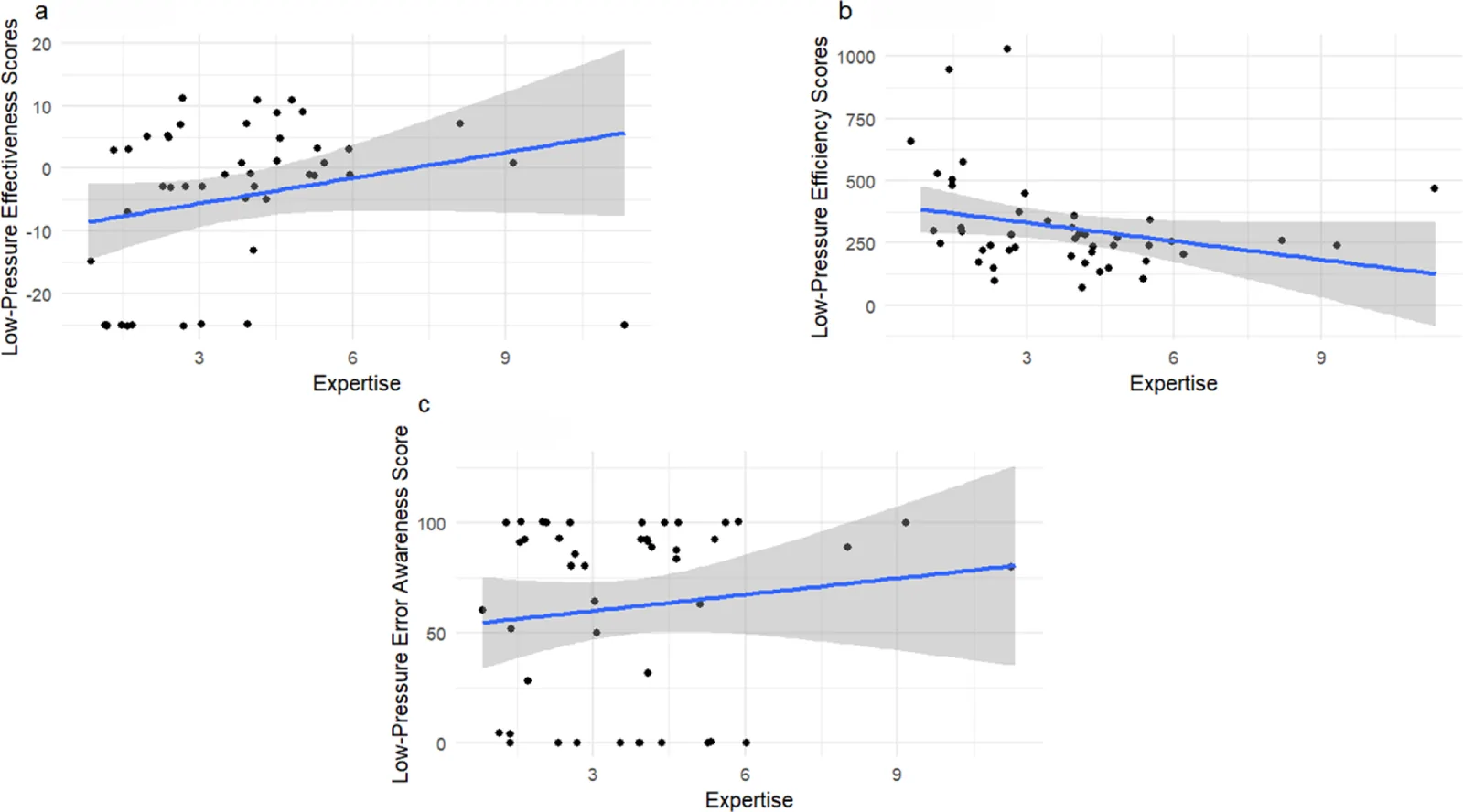
Shows the relationship between sporting expertise and low-pressure effectiveness scores (a), low-pressure efficiency scores (b), and low-pressure error awareness score (c)

### Does Expertise Predict Greater Response Inhibition Effectiveness, Response Inhibition Efficiency, and Cognitive Inhibition (i.e., Error Awareness) at High-Pressure (H2)

Within the high-pressure condition, expertise significantly predicted both response inhibition effectiveness (*F* (1, 43) = 7.29, *p* = .010, β = .38, 95% CI = [.10, .67]; see Figure 4(a)) and efficiency (*F* (1, 43) = 4.99, *p* = .031, β = −.32, 95% CI = [−.61, −.03]; see Figure 4(b)). The *R*^*2*^ values suggested the expertise accounts for 14.5% (*R*^*2*^ = .144) of the variance in effectiveness and 10.4% (*R*^*2*^ = .104) of the variance in efficiency in the high-pressure condition. However, expertise was not a significant predictor of cognitive inhibition (i.e., error awareness) in the high-pressure (*F* (1, 43) = .85, *p* = .360, β = .14, 95% CI = [−.16, .44]; see Figure 4(c)) condition. The *R*^*2*^ values suggested that expertise accounted for 1.9% (*R*^*2*^ = .019) of the variance in cognitive inhibition in the high-pressure condition. These results provide partial support for H2 in that expertise appears to be relevant for response inhibition at high-pressure, but not cognitive inhibition (i.e., experts are no more or less able to recall errors than novices).

**Figure 4.**
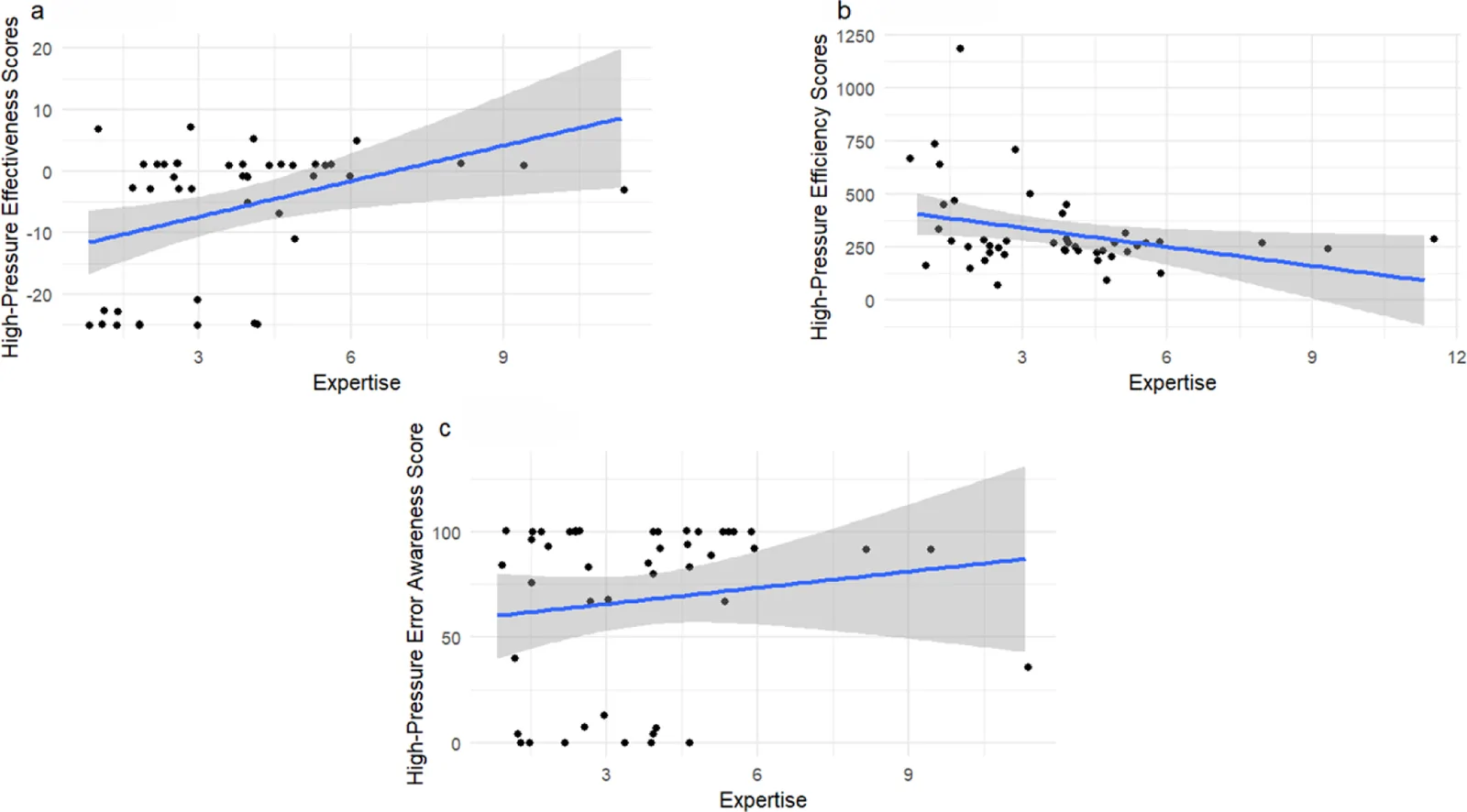
Shows the relationship between sporting expertise and high-pressure effectiveness scores (a), high-pressure efficiency scores (b), and high-pressure error awareness score (c)

### Does Higher Expertise Allow for Comparable or Improved Response and Cognitive Inhibition at High-Pressure Compared to Low-Pressure (H3)

Expertise did not significantly predict effectiveness change scores (*F* (1, 43) = .89, *p* = .352, β = .14, 95% CI = [−.16, .45]; see Figure 5(a)), efficiency change scores (*F* (1, 43) = .17, *p* = .682, β = −.06, 95% CI = [−.37, .24]; see Figure 5(b)), nor error awareness change scores (*F* (1, 43) = .01, *p* = .967, β = .14, 95% CI = [−.16, .44]; see Figure 5(c)) which suggests that performance changes from low-to high-pressure conditions were independent of sporting expertise. The *R*^*2*^ values from the individual regressions suggested that sporting expertise accounted for 2% (*R*^*2*^ = .020) of the variance in effectiveness, 0% (*R*^*2*^ = .004) of the variance in efficiency, and 2% (*R*^*2*^ = .015) of the variance in error awareness. The linear regressions allow us to comment on whether expertise may facilitate improvements under pressure however it was also hypothesised that performance may be comparable in experts (i.e., change scores at or near zero) which cannot be easily detected in regression analyses. However, inspection of Figure 5(a) to (c) seems to suggest that those with higher sporting expertise did not tend to cluster around zero.

**Figure 5.**
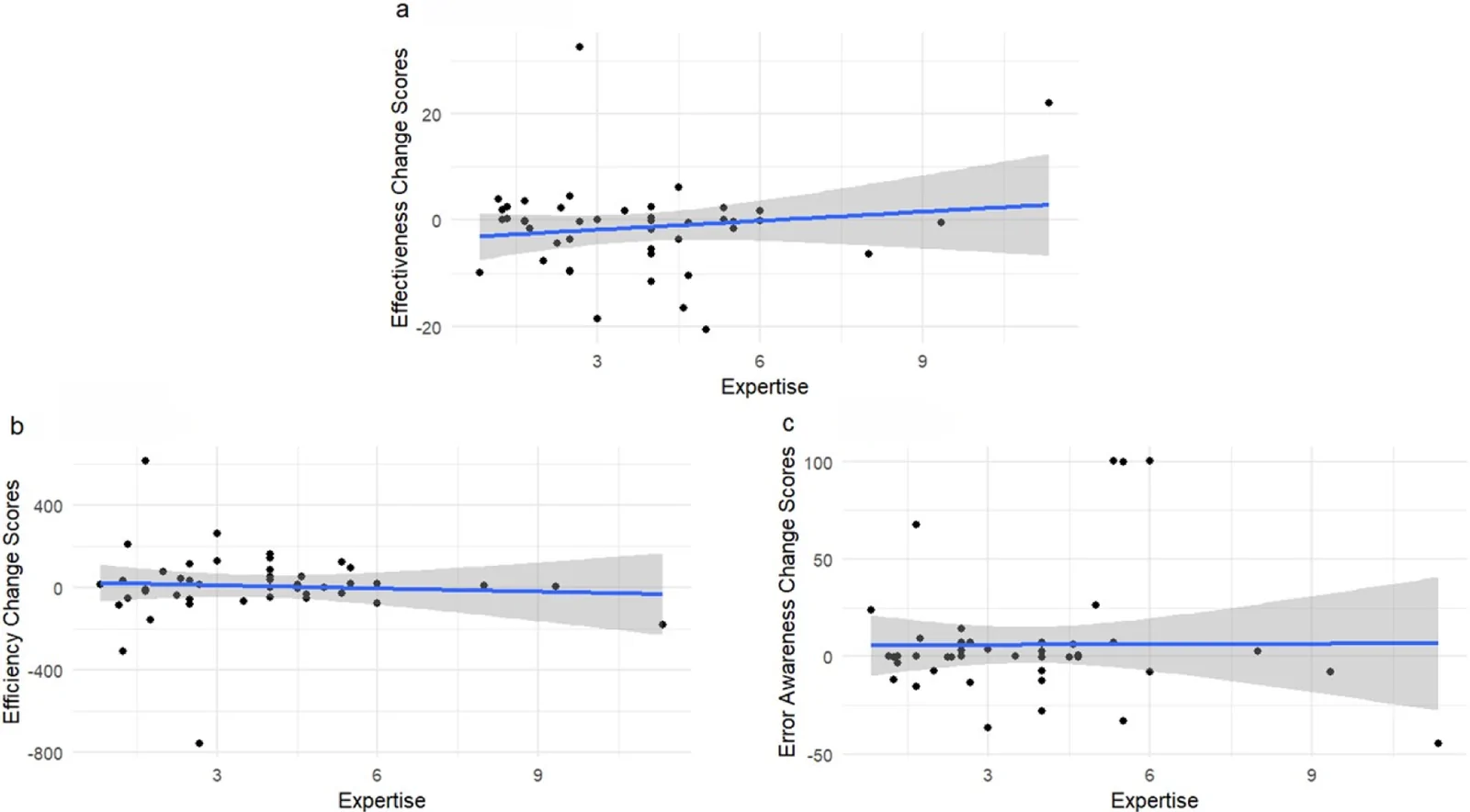
Shows the relationship between sporting expertise and effectiveness change scores (a), efficiency change scores (b), and error awareness change scores (c)

### Exploratory Analysis

Harris et al. (2019) used real-world NFL data to showcase that errors are more likely at high-pressure, and making an error increases the likelihood of subsequent errors in the moments that proceed. However, this notion is relatively untested in experimental work with athletes. This exploratory analysis was conducted using logistic regression to understand whether an error on the SST increased the likelihood of the following trial resulting in an error and whether this effect was more prominent at high-pressure. For this analysis, trials were first coded either as *0* for correct or *1* for error. Next, a new variable was created where trials were marked as *0* for post-correct trials and *1* for post-error trials. This new post-error variable was entered as the predictor while accuracy on the post-error trial (*0* = incorrect and *1* = correct) was the dependent variable. The code for the exploratory analysis is available via the Open Science Framework: https://osf.io/xg3vd/?view_only=81b1f402b28a42ca978b08ee0b97e164.

Across the 45 participants, all of whom completed two test blocks, there was a total of 10,512 trials within the SST that could be included in the logistic regression. 8,310 trials were correct, and 2,202 trials were errors. Regarding the post-error trial count, 8,315 were post-correct trials and 2,197 were post-error trials. Next, we examined how the post-error status of the trial impacted the count of correct and incorrect responses. There were 1,368 trials that were an error following a correct trial, 6,947 trials that were correct following a correct trial, 834 errors following an error trial, and 1,363 correct responses following an error trial. This is visualised in Figure 6(a). The results of the logistic regression suggested that post-error trial was a significant predictor of accuracy (*b* = −1.13, SE = .05, *p* < .001) and showed that committing an error significantly decreased the likelihood of success on the following trial. The odds ratio was .32, indicating the likelihood of an accurate response after an error are reduced by 68%.

**Figure 6.**
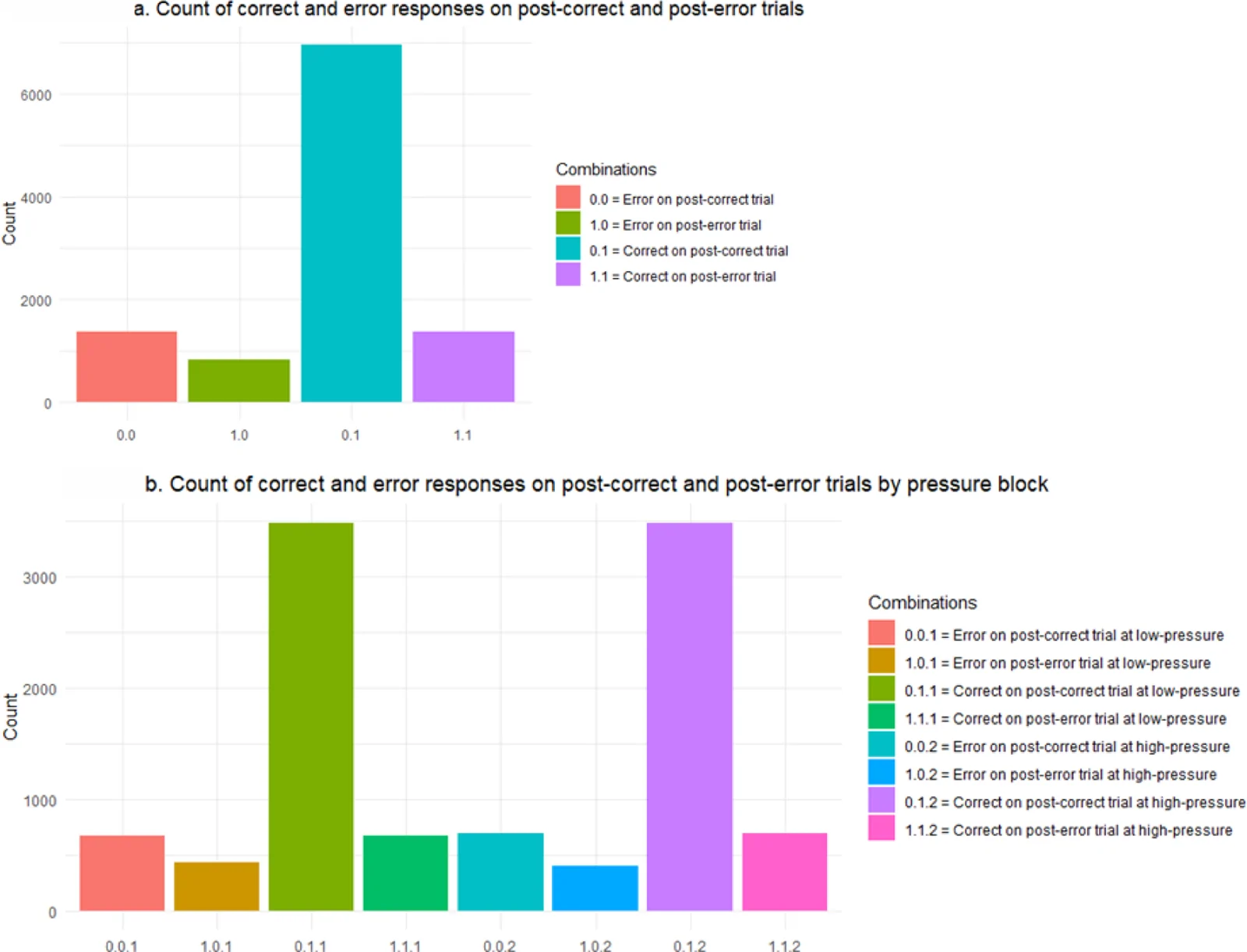
Shows the distribution of errors on post-correct, errors on post-error, correct on post-correct, and correct on post-error trials across the stop signal task (a) and the distribution of errors on post-correct, errors on post-error, correct on post-correct, and correct on post-error trials by pressure condition (low-pressure and high-pressure) across the stop signal task (b)

Finally, we examined how the pressure condition impacted post-error trial accuracy. As shown in Figure 6(b), there was a somewhat equivocal distribution across pressure conditions suggesting pressure did not seem to lead to an increase in post-error trial mistakes, as predicted and outlined in Harris et al. (2019). This idea was corroborated by the logistic regression which showed that the post-error trial and pressure condition interaction was a non-significant predictor of accuracy (*b* = .16, SE = .11, *p* = .140).

## Discussion

The aim of the present study was to understand the predictive capacity of sporting expertise upon the typically examined area of inhibition - response inhibition (i.e., effectiveness and efficiency) and the less understood area of cognitive inhibition (i.e., error awareness via an expertise-induced-amnesia like state). Moreover, based on the work of Harris et al. (2019), we wanted to examine whether sporting expertise was more poignant for performance at levels of high-pressure compared to low-pressure. The results offered partial support for our hypotheses. Regarding H1, sporting expertise did not appear to predict response inhibition effectiveness, efficiency, or cognitive inhibition at low-pressure. We found partial support for H2 as sporting expertise did predict both response inhibition effectiveness and efficiency at high-pressure, but not cognitive inhibition. Finally, H3 was not supported as expertise did not lead to comparable or improved effectiveness, efficiency or error awareness at high-pressure compared to low-pressure. Interestingly, our exploratory analyses support some ideas from Harris et al. (2019) in that trials that immediately followed an error were more likely to be an error themselves. However, this likelihood of successive error was not increased at high-pressure. The findings are interesting for the future of response and cognitive inhibition and are discussed below.

Individuals with higher sporting expertise had improved effectiveness and efficiency scores on the SST suggesting improved response inhibition. However, the relationship was not quite so straightforward. That is, in the low-pressure condition, sporting expertise did not predict effectiveness or efficiency. The lack of effect at low-pressure may be because all participants felt comfortable (i.e., low anxiety or low stress) and were able to function successfully (Eysenck et al., 2007). This is somewhat corroborated by the non-significant change in SRQ responses from baseline to low-pressure suggesting that, overall, people did not feel stressed and likely did not suffer from disrupted inhibition or goal-directed activity that is normally associated with feelings of stress/anxiety/pressure (Corbetta & Shulman, 2002). Indicating that in low-pressure situations novice and expert response inhibition performance may be more aligned. This is a novel finding in this area as research on EF often omits low- and high-pressure conditions (e.g., Hagyard et al., 2021) and therefore, lacks the detail to make this distinction.

Though sporting expertise was not a predictor at low-pressure, higher expertise significantly predicted both effectiveness and efficiency in the high-pressure condition. This is, on face value, more aligned with the previous research into expertise differences in response inhibition. For example, Elferink-Gemser et al. (2018) showed that elite table tennis players make fewer errors on a colour-word interference task than sub-elite players, Vestberg et al. (2012) outlined that high-division youth soccer players had better behavioural inhibition than low-division youth soccer players, and Hagyard et al. (2021), who treated expertise as a continuous variable, found a positive association between sporting expertise and performance on the SST. The addition here is that we can now specify that expertise is advantageous for improving response inhibition ability uniquely under high-pressure. It’s important to note, though, that the sample comprised athletes from various sport types which introduces variance in training hours, cognitive and attentional load, and motor demands. These factors could have acted as confounds, not accounted for in our work, that could explain the pattern of inhibition performance beyond generic expertise levels in the present work. Future work should obtain a larger sample to allow for sport type analyses.

Figures 3 and 4 highlight an interesting finding. Specifically, Figures 3(a) and 4(a) show that a number of participants, typically scoring around the lower end of the expertise scale, showed very low effectiveness scores in both the low- and high-pressure conditions. Given this is a somewhat consistent finding, the clustering near the bottom of the scale may be an indicator of task difficulty as opposed to random error. The SST is a cognitively challenging task that individuals lower in expertise may have struggled to inhibit their responses on. This pattern is also consistent with the idea of a floor effect where it’s difficult to discriminate between performers at the lower end of the scale as scores are compressed near the minimum value. This pattern may contribute to the non-significant findings at low-pressure levels and future work may wish to utilise easier or more intuitive (e.g., a sport-specific task for athletes) tasks. It should be noted here that the “stop-signal” delay was adjusted based on performance (i.e., increased by 50 ms on successful inhibitory trials and decreased by 50 ms on unsuccessful trials) to try and accommodate for ability (as per Verbruggen et al., ’s 2019 recommendations) though this may not have been enough.

The results pertaining to cognitive inhibition were unexpected. Those with more expertise did not report significantly fewer errors as we hypothesised and as could potentially be expected from the expertise-induced-amnesia supposition (Beilock & Carr, 2001). This could simply be because the initial application for expertise-induced-amnesia was to motor control and thus, is not relevant for cognitive processes. Another explanation could be that the expertise-induced-amnesia is relevant only to the well-learnt or ‘perfected’ skills of athletes and the current task was novel with little chance to achieve ‘expertise’. The lack of ecological validity for athletes in the current task also supports this and perhaps efforts should be made to assess cognitive inhibition within a domain related to the athlete. Although, these are somewhat contradictory to our expectations and that of recent research (e.g., Harris et al., 2019).

Instead, it could be that the task instructions promoted participants to remember errors. In our instructions we emphasised that a recalled error should be marked with double key press on the following trial. In doing so, we may have inadvertently drawn extra attention to this and made it seem more pertinent to remember errors rather than natural memory responses. This could also be supported by some of the previously cited research that suggest experts have better attentional control (e.g., Hagyard et al., 2021). The caveat to this, if experts had significantly better attention to errors, or followed task instructions more closely, then they should have recalled significantly *more* errors, which was also not the case. Further research is encouraged to take our available task code and look to improve and find a more natural way of detecting error awareness in athletes (e.g., ask individuals to recall all errors from the block at the end of each block). This raises another issue with how individuals were asked to provide error awareness. Our double key press approach was not validated as a measure of error awareness and thus may not have been the most suitable way to capture the construct.

Another explanation could come from neuropsychology. The free energy principle is a neuropsychological brain theory which outlines that adaptive systems (e.g., the human brain) look to minimise uncertainty and thus, optimise value (i.e., ‘reduce free energy’; Friston, 2010). In such accounts, uncertainty is accompanied by substantial metabolic outlays that arise because our predictions are inaccurate (and thus, the situation is uncertain; Friston, 2010). A rapid metabolic shift or reaction to this uncertainty can bring about increased interpretations of anxiety that indeed may be linked to poorer task performance in the subsequent moments (as outlined in Harris et al., 2019). It may be that experts do not ‘forget’ their mistakes as per an expertise-induced-amnesia model (Beilock & Carr, 2001) and instead find mistakes less ‘surprising’ or are perhaps better analysers of their mistakes (i.e., less surprised about their origin). Due to the lack of surprise, metabolic cost and subsequent anxiety about the trial is reduced. The fact that individuals with higher expertise will almost certainly experience mistakes across their time within the sport makes each individual mistake feel less of an issue (i.e., less surprising) and instead, allows the expert to continue performing at a maintained or enhanced level. Indeed, expertise was linked with improved effectiveness and efficiency under high-pressure, just not error awareness.

Finally, predictions around H3 were not accurate. That is, expertise was not associated with equivocal (indexed through change scores near zero) nor improved (indexed by enhanced change scores) performance in effectiveness, efficiency, or error awareness at high-pressure compared to low-pressure. This is unusual as we anticipated that experts would show no deterioration in performance or even show enhanced performance as per models of ‘clutch’ performance (Otten, 2009), compared to those with less expertise. This is somewhat contradictory to the significant findings associated with H2 in that, if expertise was important for greater response inhibition at high-pressure how come expertise was not also a marked of maintained or improved change scores. One explanation could be that change scores are not a sensitive enough outcome measure to address H3. Though not theoretically aligned to the current hypothesis, it may be better for future work to obtain a larger sample size so that mixed-effect models can be utilised. Such an approach allows for interaction effects across conditions and may be more sensitive to differences in performance across low- and high-pressure conditions.

It could also be that the pressure instructions did not fully achieve their intended effect. Although there were significant increases in SRQ scores between low- and high-pressure conditions, the associated effect size was small suggesting only a modest effect of the manipulation. Indeed, a similar lack of effect of pressure manipulations has been shown to not evoke the described effect in previous research into EFs in athletes (Brimmell et al., 2021). It could also be that alongside the increase in SRQ responses individuals recruited additional resources to cope with the demands of the stressful situation. One potential resource pertinent to research (e.g., Edwards et al., 2017) and theory (i.e., Attentional Control Theory; Eysenck et al., 2007) is effort. It may be that individuals were able to increase the effort they put into the task in order to maintain performance. This can be somewhat verified by the information shown in Figure 5 as they show that change scores were typically always around zero, irrespective of expertise score. Potential positive resources like effort, or motivation, could be included in subsequent research as covariates.

A brief exploratory analyses was conducted to examine the interesting idea that errors can lead to more errors in subsequent moments. This idea stems from research using real-world data on NFL plays (Harris et al., 2019) and tennis matches (Harris et al., 2021). Yet, there is little experimental work that has examined this prospect within a controlled environment. The exploratory analysis partially supported previous work in that an error on the SST was significantly more likely to lead to an error on the next trial within the SST. However, we did not find this result to be more prominent in the high-pressure condition as Harris et al. (2019) outlined the relationship would be. Rather than suggest pressure does not increase the likelihood of perpetual errors, this finding could have arisen for similar reasons that the change scores were not related to sporting expertise. That is, the recruitment of additional resources like effort or motivation could combat the self-fulfilling prophecy of continued mistakes within the SST at high-pressure.

One possible theoretical explanation for this is processing efficiency theory (Eysenck & Calvo, 1992). This theory suggests that the participants will recruit additional resources namely an increase in effort and motivation towards the task; the increase in these two resources is due to the presence of cognitive anxiety and used in combating what might be interpreted as poor performance in a task (Hardy et al., 2007). This would allow the participants in this study to compensate when making a mistake by applying the extra resources. One key point to note is that performance will be increased if the perception of success is at a suitable level meaning a performer is confident of success in the task (Eysenck, 2012). Again, within this study it could be that participants feel they have a good chance of being successful at the task if they were simply to apply more resources whilst under pressure.

### Limitations

The present study has several limitations. First, the classification of expertise using Swann et al.’s (2015) model should be interpreted cautiously. This model only allows for classification at ‘elite’ levels with ‘semi-elite’ being the lowest category. This is problematic here as the majority of our participants scores came from playing a competitive sport and years experience which alone don’t seem sufficient to be called ‘elite’. Here, we followed the model but urge future research to build a model more inclusive of athletes across the performance spectrum (e.g., amateur to elite). Also, the ecological validity of the task can be questioned. The inhibition task was selected given its wide-spread use and established ability to tap inhibition ability (Verbruggen et al., 2019). However, it is void of any sporting context and thus, falls into similar issues of previous research concerned with how these findings can translate to real-world performance and whether the tasks are really relevant and key for sporting populations. Future work is encouraged to develop and utilise sport-specific tasks of executive functions like inhibition which would allow for the influence of sporting category (i.e., static, interceptive, strategic) to be explored in more naturalistic tasks.

Another potential limitation with the selected task is that methods of measuring cognitive inhibition are limited and thus the authors here attempted to operationalise the task and outcome measures based on personal applications of the theorised model of expertise-induced-amnesia (Beilock & Carr, 2001). It may be that any findings associated with error awareness were due to the task not doing as intended. Future researchers are encouraged to adapt this task and think about novel ways to assess how athletes may be able to move beyond errors and win points/matches/championships when it matters most. Another potential limitation is the fixed order used in the pressure manipulation. The pressure manipulation procedure replicates previous methods used in the field (Brimmell et al., 2021) and are based off literary recommendations (Gropel & Mesagno, 2019). However, the fixed order (i.e., low then hight) may result in order and practice effects influencing the findings. Future research should consider pressure manipulation designs that allow for counterbalancing. In addition, future research could consider using behavioural and physiological measures (e.g., heart rate, pupil diameter) to enhance validity.

## Conclusion

The present study was the first to examine expertise as both a predictor of response inhibition (effectiveness and efficiency) and cognitive inhibition (error awareness) in both low- and high-pressure conditions. Sporting expertise does not appear to influence response inhibition at low-pressure, but sporting expertise is an important, and significant, factor for good response inhibition under high-pressure. Sporting expertise does not make athletes any less (as hypothesised) or more able to forget or recall their errors. Change scores were calculated between the low- and high-pressured conditions to see how performance changed on the SST task. Expertise was unable to predict these change scores suggesting performance was similar despite the pressure condition. In sum, response inhibition is important for expert performance under high-pressure and we need more research to understand how experts move past errors and maintain task focus.

## Note

1 To calculate SSRT the *n*th reaction time is found by multiplying the probability of responding on “stop-signal” trials by the total number of “go” trials. This creates the *n*th value which is matched to the corresponding reaction time from the complete distribution of “go” trial reaction times to give the *n*th reaction time. The average stop signal delay across all “stop-signal” trials is then taken away from the *n*th reaction time to give the SSRT. See Verbruggen et al. (2019) for a full description.
